# Prostate Cancer—PET Imaging Update

**DOI:** 10.3390/cancers15030796

**Published:** 2023-01-28

**Authors:** Sankarsh Jetty, James Ryan Loftus, Abhinav Patel, Akshya Gupta, Savita Puri, Vikram Dogra

**Affiliations:** 1 Department of Imaging Sciences, University of Rochester Medical Center, New York, NY 14642, USA; 2 Department of Radiology and Imaging Sciences, Indiana University School of Medicine, Indianapolis, IN 46202, USA

**Keywords:** PET, prostate cancer, PSMA, Axumin, biochemical recurrence

## Abstract

**Simple Summary:**

The imaging of prostate cancer has evolved over the last few decades with molecular imaging, especially Positron Emission Tomography (PET) replacing conventional CT and MRI in patients with prostate cancer recurrence. These recent advances in PET imaging have allowed for a better localization of disease recurrence, and a better characterization of disease extent. Beyond localization and characterization, new uses for PET imaging have emerged, with PET agents now playing a role in assessing the response to treatment at the receptor level, and also helping to determine patient prognosis and survival.

**Abstract:**

Prostate cancer is the most common non-dermatologic cancer in men, and one of the leading causes of cancer-related mortality. The incidence of prostate cancer increases precipitously after the age of 65 and demonstrates variable aggressiveness, depending on its grade and stage at diagnosis. Despite recent advancements in prostate cancer treatment, recurrence is seen in 25% of patients. Advancements in prostate cancer Positron Emission Tomography (PET) molecular imaging and recent United States Food and Drug Administration (FDA) approvals have led to several new options for evaluating prostate cancer. This manuscript will review the commonly used molecular imaging agents, with an emphasis on Fluorine-18 fluciclovine (Axumin) and PSMA-ligand agents, including their protocols, imaging interpretation, and pitfalls.

## 1. Introduction

Prostate cancer is the most common non-dermatologic cancer in men, and the second leading cause of cancer-related death. The incidence of prostate cancer increases with patient age, with an incidence rate of 1 in 350 men for those under 50 years of age, climbing to an incidence of 60% in men over the age of 65. Apart from age, additional risk factors for developing prostate cancer include African-American ethnicity, lower socioeconomic class, diets rich in saturated fat and poor in vegetables, and a family history of prostate cancer [1]. Radical prostatectomy and radiation therapy are two primary avenues of treatment with curative intent, and they remain the most common treatment choices in patients with organ-confined prostate cancer. Additionally, for patients with metastatic castration-resistant prostate cancer, a new theranostic gamma and beta-emitting agent, Lutetium-177 Prostate-Specific-Membrane-Antigen-617 (Lu-177-PSMA-617, Pluvicto), has been shown to improve progression-free and overall survival [2]. The aim of this article is to provide an overview of prostate cancer molecular imaging agents, including their use for initial diagnosis, locoregional, and metastatic disease/recurrence, as well as to provide insights into exciting new developments for the future of prostate cancer imaging.

### 1.1. Biochemical Recurrence of Prostate Cancer

**Figure 1:**
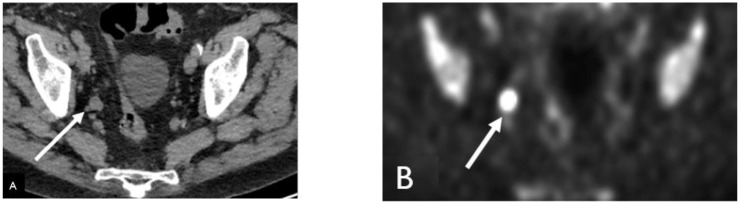
Sixty-nine-year-old male with history of prostate cancer, status post-prostatectomy and salvage radiation, now with rapidly rising PSA. Non-contrast-enhanced-CT (NECT), (**A**) shows an enlarged right pelvic side wall lymph node. Corresponding Fluorine-18 Fluciclovine (Axumin) PET image (**B**) shows intense radiotracer uptake concerning for recurrence and nodal disease.

Approximately one-third of men undergoing definitive treatment for prostate cancer have subsequent relapse [3]. Conventionally, cancer recurrence is discovered and monitored using prostate-specific antigen (PSA). Biochemical recurrence is seen in 25% of patients, defined as a rise of PSA to >0.2 ng/mL following radical prostatectomy, or an increase of 2 ng/mL or more following radiation [4]. Several risk factors for prostate cancer recurrence have been identified, including initial preoperative PSA value, seminal vesicle involvement, and positive surgical margins after radical prostatectomy [5]. Understanding the sites and extent of disease recurrence has a substantial role in determining treatment options (Figure 1). Nuclear medicine, computed tomography (CT), and magnetic resonance imaging (MRI) exams all play a role in determining local and metastatic disease recurrence. However, newer Positron Emission Tomography (PET) agents have been developed to increase the sensitivity and specificity, and to improve the localization of prostate cancer recurrence.

### 1.2. Earlier Generation Gamma-Emitting Planar/SPECT Agents

**Figure 2:**
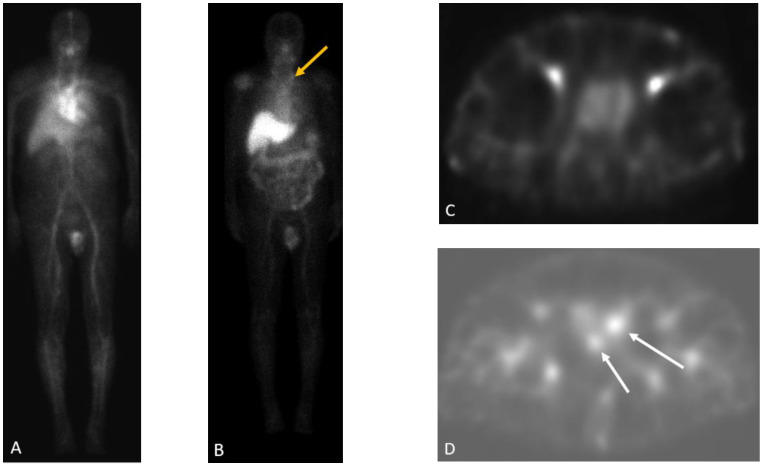
Sixty-four-year-old man with history radical prostatectomy for prostate cancer (Gleason 4 + 4) with rising PSA. In111 capromab pendetide whole body blood pool (**A**) and 96 h delayed (**B**) images demonstrate a focus of increasing radiotracer activity in the left neck base corresponding to a metastatic supraclavicular (Virchow) node (yellow arrow in **B**). SPECT blood pool (**C**) and delayed (**D**) images of the pelvis demonstrate two foci of increasing radiotracer activity in the right and left prostate bed, compatible with local recurrence (white arrows).

Indium-111 (In-111) capromab pendetide (ProstaScint^®^) was the earliest FDA-approved PSMA-ligand radiotracer. In-111 capromab pendetide is made up of In-111 attached to a murine monoclonal antibody termed 7E11C5.3, which binds to the cytosolic domain of PSMA [6,7]. While binding to the cytosolic domain of PSMA is thought to lead to greater specificity, there is concern about the ability to bind viable cells with intact membrane; however, this has been demonstrated in vitro [8]. In-111 is a medium-energy gamma-emitting agent with photopeaks at approximately 171.3 and 245.4 kEv, and a half-life of approximately 67.2 h. The recommended dose of In-111 capromab pendetide is 185 MBq (5mCi), with an estimated total body radiation dose of 27 mGy [7]. Imaging is performed at 30 min and between 72 and 120 h, following injection with whole-body planar and single photon emission computed tomography (SPECT) or SPECT/CT images of the pelvis at minimum. Hybrid SPECT/CT provides for more robust attenuation correction, target-to-background ratio, and increased confidence in anatomic localization, which has been shown to lead to an improved detection of local recurrence and the alteration of management in a large portion of patients (up to 78%), compared to planar imaging [9]. For the delayed images, the patient should be prepped with a cathartic and enema to reduce bowel activity. Physiologic distribution includes prostate tissue, thought to be upregulated in prostate cancer, and other non-androgen-dependent sites including salivary epithelial tissue, liver, spleen, bone marrow, and bowel with urinary excretion [7]. Blood pool activity predominates on the early phase images, and it should decrease upon delayed imaging. Some groups advocate for blood pool activity to be imaged with a dual photo peak technique, with Technetium-99m (Tc-99m)-labeled red blood cells at the time of delayed imaging, rather than the In-111 blood pool images [10]. Positive findings representing prostate malignancy should show increasing relative activity on the delayed scans owing to intra-cytosolic binding of the radiotracer, and clearance of the blood pool (Figure 2). There are abundant pitfalls of non-prostatic disease demonstrating radiotracer activity, including degenerative arthropathy, tortuous vascularity, and infectious and inflammatory conditions of the bowel. Additionally, the interpreter must be aware of the potential of a human anti-mouse antibody response (HAMA), reported in approximately 8% of patients after a single dose of In-111 capromab pendetide, which can lead to errors in interpretation due to elevated liver and bowel activity [7]. In the initial staging, In-111 capromab pendetide has been reported to have up to a sensitivity of 75%, a specificity of 86%, and an accuracy of 81% for the detection of extra-prostatic malignant disease in high-risk patients undergoing radical prostatectomy with pelvic lymph node dissection [11]. For patients with biochemical recurrence following radical prostatectomy, one study described a 76% sensitivity and a 54% specificity of In-111 capromab pendetide for assessing local recurrence, and a 69% sensitivity and 58% specificity for diagnosing distant metastatic disease [12]. Tc-99m-HYNIC-Glu-Urea-A is an additional gamma-emitting prostate specific agent under investigation at the current time. Ultimately, the utility of gamma-emitting agents in clinical practice has been and will continue to be limited, due to the poor imaging efficiency of In-111 capromab pendetide gamma emissions, the need to perform the study over multiple days, and the intrinsically greater spatial resolution and target-to-background ratio of PET agents. 

### 1.3. Prostate Targeted and Non-Targeted PET/CT Agents for Imaging

**Figure 3:**
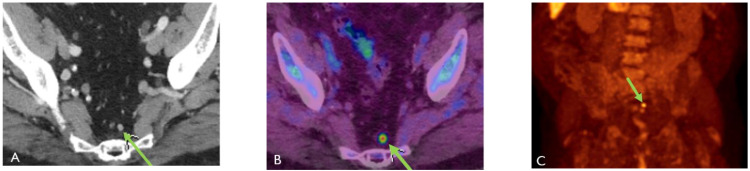
Seventy-year-old male with history of prostate cancer status post-prostatectomy, now presenting with rising PSA. (**A**) Contrast-enhanced CT (CECT) shows a normal-sized presacral lymph node which was presumed to be reactive. Axumin-fused PET/CT (**B**) and MIP (**C**) images show Axumin uptake within the lymph node, suspicious for recurrence and nodal disease.

Historically, computed tomography (CT) and magnetic resonance imaging (MRI) rely on lymph node size and morphology instead of function to detect nodal metastasis. They have poor sensitivity, detecting less than 40% of nodal disease when a 10-mm size cutoff in the short axis is used (Figure 3) [13]. Due to this limitation of anatomic imaging and gamma-emitting agents, various PET tracers have been developed and used for prostate cancer imaging, particularly for staging and suspected recurrence/metastatic disease [14]. First, Carbon-11 Choline (C-11 Choline) was first approved in 2012 by the Food and Drug Administration (FDA) for imaging men with a biochemical recurrence of prostate cancer. Next, Fluorine-18 Fluciclovine (Axumin) was approved by the FDA in May of 2016 for the same indication. While these were a significant upgrade to the previous imaging standard for prostate cancer, research continued on prostate-targeted PET agents, and in December of 2020, the FDA approved Gallium-68 PSMA-11 (Ga-68 PSMA-11, Illuccix, Locametz). Ga-68 PSMA-11 was found to be significantly better at detecting prostate cancer recurrence than Axumin in patients with early biochemical recurrence after prostatectomy, with the exception being locoregional recurrence where Axumin was more sensitive due to its lack of radiotracer in the bladder [15,16]. Finally, in May of 2021, the FDA approved 18F-fluoro-pyridine-3-carbonyl)-amino]-pentyl}-ureido)-pentanedioic acid (DCFPyL, Pylarify), which conferred several advantages over Ga-68 PSMA-11, including a longer half-life and a lower positron energy level, leading to improved count statistics and spatial resolution [17,18].

### 1.4. C-11 and F-18 Choline

Choline is a precursor for the synthesis of phospholipid phosphatidylcholine, a component of the cell membrane. Once it enters the cell, it becomes phosphorylated into phosphorylcholine by choline kinase and is trapped within the cell [19]. Cancer cells, including prostate cancer cells, demonstrate increased cell membrane synthesis. Choline imaging exploits this with imaging, demonstrating areas of choline uptake corresponding to areas of cancer cells. Two radioactive tracers are traditionally used in imaging with choline, Carbon-11 (C-11) and Fluorine-18 (F-18). The key differentiating factor between the two tracers is their half-life, with C-11 Choline having a much shorter half of 20 min compared with 110 min for F-18 Choline. The normal distribution of C-11 Choline and F-18 Choline is similar, with high levels of activity seen within the liver, spleen, kidneys, pancreas, and salivary glands. Activity is also seen within the bone marrow and bowel [20,21]. One disadvantage of F-18 Choline is that it has a higher amount of tracer activity within the renal excretory system, confounding the visualization of radiotracer uptake in the prostate and lymph nodes along the course of the ureters [22]. As such, C-11 Choline is preferred, and is the only one of the two agents approved for use in the United States. Given its short half-life; however, an on-site cyclotron is required. Another downside of Choline PET in general is that its uptake is non-specific and can be seen with other disease processes, including benign entities such as benign prostatic hyperplasia and malignant, non-prostate causes such as invasive thymoma, Non-Hodgkin Lymphoma, and renal cell carcinoma [23,24,25]. Despite this, Choline performs better than conventional imaging when evaluating prostate cancer recurrence, with a detection rate of 62% [26]. F-18 Choline was also found to have a sensitivity and specificity of as high as 93% and 91%, respectively, in prostate cancer patients with biochemical recurrence; however, F-18 PSMA had a higher detection rate [27].

### 1.5. Fluorine-18 Fluciclovine

Fluorine-18 Fluciclovine (Axumin), or anti1-amino-3–18F-Fluorocyclobutane-1-carboxylic acid (FACBC), was an immediate improvement to the standard of care for prostate cancer imaging at the time of its approval. It was approved by the FDA specifically for the imaging of prostate cancer recurrence following treatment [28]. Axumin is a radiolabeled analog of levorotary leucine, an essential amino acid. It takes advantage of the increased level amino acid transport in prostate cancer cells by being taken up via the human L-type amino acid transport and alanine–serine–cysteine transporter systems. These transporters are typically upregulated in many carcinomas, especially prostate cancer. Axumin does not undergo metabolism and can transit through the channels that it enters [29].

### 1.6. Gallium-68 Prostate-Specific-Membrane-Antigen-11

Gallium-68 Prostate-Specific-Membrane-Antigen-11 (Ga-68 PSMA-11) was the first prostate-specific PET agent approved by the FDA for evaluating prostate cancer recurrence and metastatic disease. Additionally, it is generally the agent that is used in determining patient eligibility for Lu-177 PSMA-617 therapy [2]. Prostate-specific membrane antigen (PSMA) is a peptidase that works to hydrolyze N-acetyl-L-aspartyl-L-glutamate (NAAG) into N-acetyl-L-aspartate (NAA), and L-glutamate and poly-y-glutamated folate into mono-glutamylated folates [30]. It is expressed as a cell surface protein, and is 10 to 80 times more abundant in prostate cancer cells [31,32]. Non-prostate malignant uptake has been seen with several malignancies, including hepatocellular carcinoma, renal cell carcinoma, and breast cancer [32].

### 1.7. Fluorine-18 DCFPyL 

18F-fluoro-pyridine-3-carbonyl)-amino]-pentyl}-ureido)-pentanedioic acid (DCFPyL) or Fluorine-18 Piflufolastat (Pylarify) is the newest PET imaging agent, and it combines the accuracy of PET with the precision of PSMA targeting to identify PSMA-avid loco-regional disease and metastatic disease. There are several advantages to Fluorine-18 over Gallium-68 [17,18].

- Fluorine-18 is cyclotron-produced and is more cost-effective for mass production.
- Gallium-68′s physical half-life is shorter than Fluorine-18′s (68 min versus 110 min), limiting off-site transportation and the ability to perform delayed imaging [17].
- The positron yield of Gallium-68 is lower than Fluorine-18 (89.14% vs. 96.86%).
- Gallium-68 has higher positron energy resulting in lower spatial resolution.

### 1.8. PET Imaging Protocols

While PET use protocols vary slightly from institution to institution, the general guidelines have several principles in common.

For the administration of Axumin, it is generally recommended that patients:

- Avoid exercise for one day before the study.
- NPO for >4 h before the study.
- No voiding within 1 h of the scan (voiding can lead to early radiotracer excretion into the bladder [33]).
- Position the patient supine with arms to the side and inject 370 MBq/10 mCi, preferably into right-sided intravenous access to avoid a false positive Virchow’s node on the left.
- Flush with 0.9% normal saline.
- Reposition patients with arms above their head and scan with low-dose CT for anatomic correlation.
- Start the PET scan 3–5 min after the injection.
- At our institution, the reconstruction of fused maximum intensity projections (MIPs) are obtained in the coronal and sagittal planes, both of the entire body (vertex to knees) and of a narrow field-of-view of the pelvis.

Per the FDA, the approved indication for Axumin in PET imaging is men with suspected prostate cancer recurrence based on elevated serum PSA.

For the administration of PSMA, the above protocol can be followed with a few significant differences:

- The patient has no specific activity or NPO status requirements; however they are encouraged to drink fluids (approximately 500 mL) 2 h before the scan, to improve hydration status and the subsequent clearance of radiotracer. The patient should still void before the scan.
- After radiotracer administration, the PET scan is started for 50–100 min for Ga-68 PSMA-11, and 60–120 min for F-18 DCFPyL (60 min at our institution).

For a summary of the three PET agents, including the chemical properties and acquisition parameters, please see Table 1 [29,34,35].

### 1.9. Image Interpretation

#### 1.9.1. Axumin PET:

##### Normal Physiologic Distribution

One can compare the intensity of Axumin uptake to that of the blood pool, bone marrow, and liver, rather than relying entirely on the standardized uptake value (SUV) measurements. Avidity that is equal to or greater than that of the blood pool but less than that of the bone marrow is mild. Avidity that is equal to or greater than that of the bone marrow but less than that of the liver is moderate. Avidity that is equal to or greater than that of the liver is intense [28].

**Figure 4:**
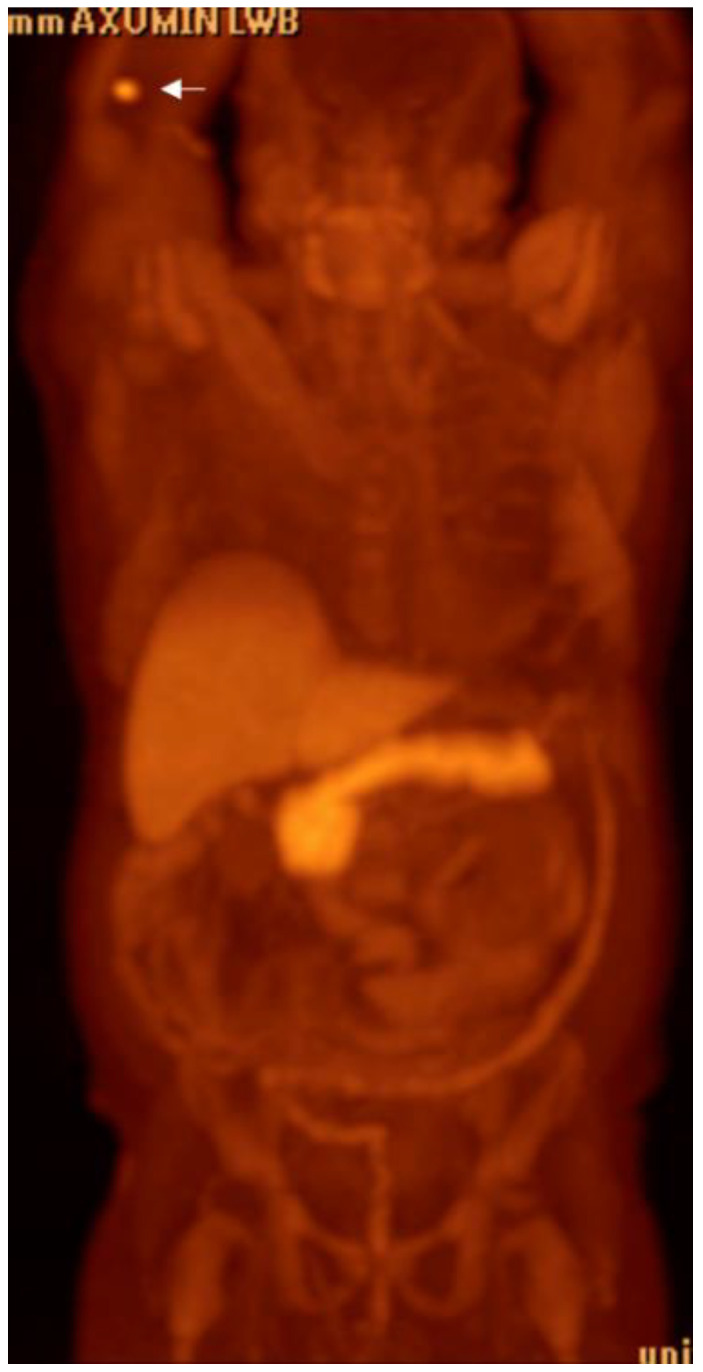
Axumin MIP image shows normal physiologic uptake within the pancreas (most intense), liver, salivary glands, small bowel, red marrow, and muscle. Note that the focus of increased uptake at the injection site in the right antecubital fossa (arrow).

Typical physiologic tracer uptake is seen within multiple areas in the body, most notably within the pancreas, liver, salivary glands, pituitary glands, small bowel, red marrow, and muscles (Figure 4). The most intense uptake is typically in the pancreas, with the dynamic uptake being the most intense within 15 min after injection, and decreasing to a lower level than the liver. The liver has the second most intense physiologic uptake and is the critical organ. The salivary glands and pituitary typically have moderate uptake, and the bowel shows variable uptake. Red marrow activity peaks at a moderate intensity 10–15 min after the injection, and decreases over time. However, muscle uptake is mild during the early phases of the examination and increases over time [34].

##### Detection of Loco-Regional Disease and Distant Metastases

**Figure 5:**
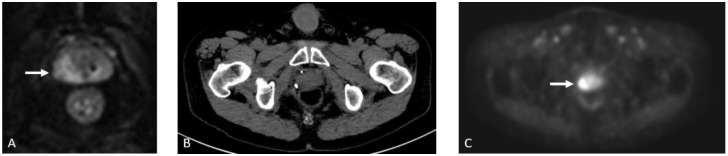
Eighty-four-year-old male with elevated PSA levels. PET image shows increased uptake within the right lateral peripheral zone concerning for recurrence. Corresponding CT shows no abnormality in the area of pathology. Magnetic resonance imaging (MRI) Diffusion-Weighted-Imaging (DWI), (**A**) sequence through the prostate shows increased DWI signal in the right mid-gland (arrow), PiRads 5. NECT (**B**) shows no discernable abnormality in the area of interest; however, corresponding Axumin PET (**C**) shows intense Axumin uptake (arrow).

**Figure 6:**
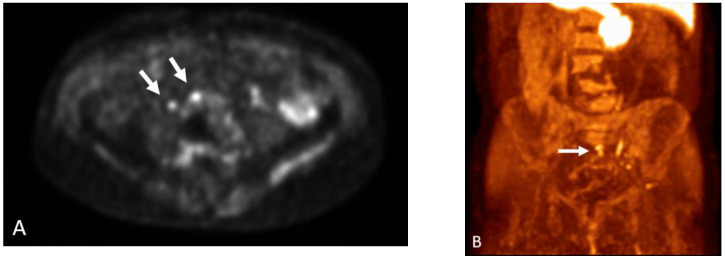
Seventy-two-year-old male with prostate cancer, status post-prostatectomy, now presenting with rising PSA levels. Axumin PET (**A**) and MIP (**B**) images show Axumin uptake within two right peri-aortic lymph nodes, suggestive of nodal disease. Also note that the abnormal uptake in the bone marrow, secondary to treatment-related effects.

**Figure 7:**
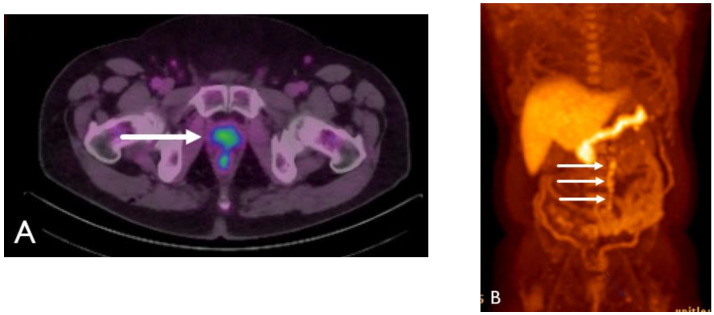
Fifty-six-year-old male with prostate cancer, status post-combination radiation and hormone therapy, now presenting with rising PSA. (**A**) Axumin-fused PET/CT image shows Axumin uptake within the prostate gland (arrow) concerning for local recurrence. MIP image (**B**) shows Axumin uptake within a chain of left peri-aortic lymph nodes (arrows) extending to the level of the kidneys.

**Figure 8:**
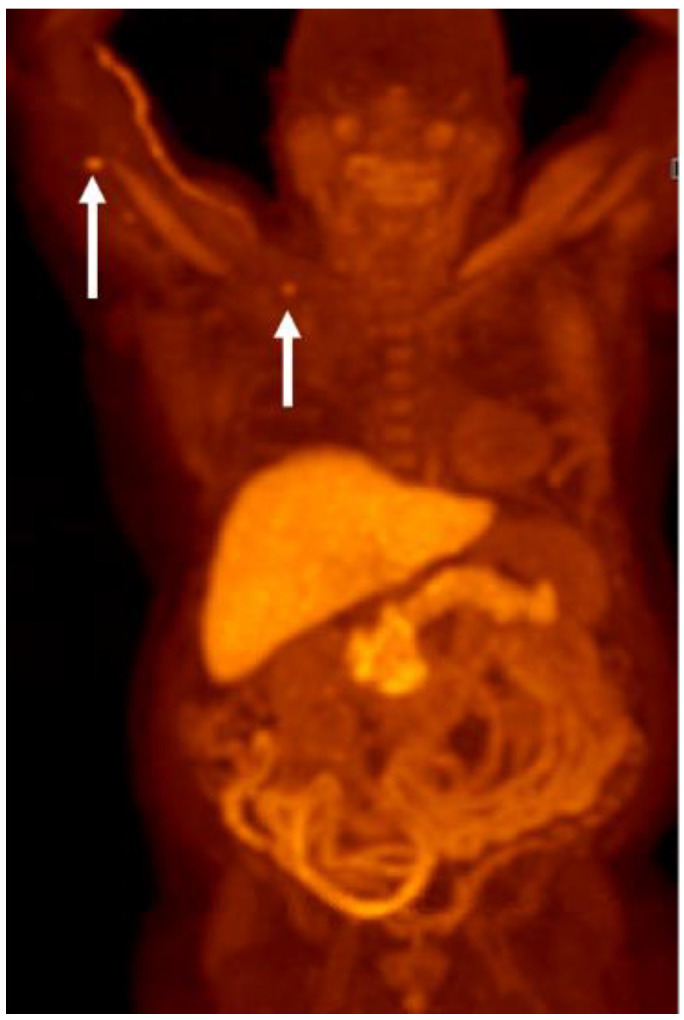
Sixty-year-year-old male with history of prostate cancer, status post-prostatectomy, now presenting with rising PSA levels. Axumin MIP image shows increased Axumin uptake in the right upper extremity and axilla (arrows) concerning for nodal metastasis.

**Figure 9:**
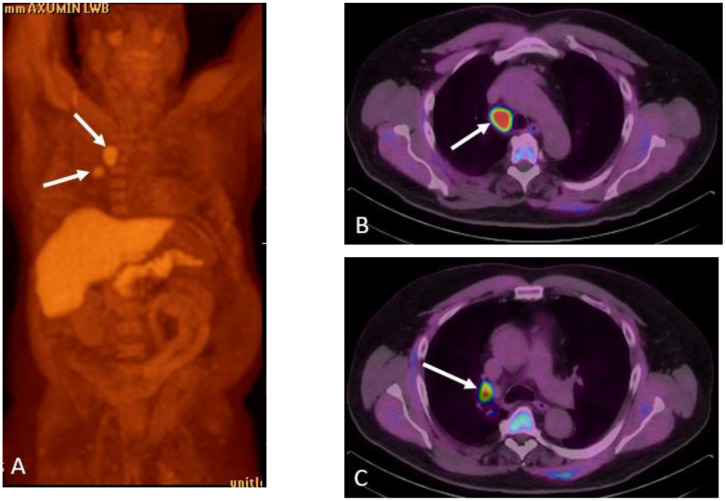
Sixty-seven-year-old man with history of prostate cancer status post-prostate, and salvage radiotherapy with rising PSA. Axumin MIP (**A**) and fused PET/CT (**B**,**C**) demonstrated right lower paratracheal and hilar lymphadenopathy (arrows) concerning for metastases from prostate cancer or a secondary lung primary. Endobronchial ultrasound fine needle aspiration demonstrated adenocarcinoma consistent with prostate primary cancer. Patient was treated with androgen deprivation therapy and localized radiotherapy with nadir to undetectable PSA.

The most common histologic subtype of prostate cancer is adenocarcinoma, which tends to arise from the peripheral zone of the prostate gland (Figure 5). As such, it is amenable to detection via digital rectal examination (DRE) [35]. At diagnosis, approximately 33% of patients present with nodal metastasis. The typical spread of regional nodal disease is to the pelvic lymph nodes, including the iliac and presacral chains progressing cranially to the retroperitoneum (Figure 6, Figure 7 and Figure 8). Unsurprisingly, the sensitivity of detection of disease is directly correlated with the patient’s PSA level. Axumin PET has a detection rate of 72.0% at PSA < 1.0 ng/mL, improving to 83.3% at a PSA between 1 and 2 ng/mL, and 100.0% above a PSA of 2.0 ng/mL [36]. More recent studies have found that the detection rate at very low PSA may be better than initially thought, with a 58% detection rate for PSA < 0.3 ng/mL, and 71.4% for PSA between 0.2 and 0.57 ng/mL [37,38]. Despite the relatively lower detection rate at lower PSA levels, Axumin PET has a negative predictive value of 100% at PSA values that are greater than 1.05 ng/mL [39]. One downside of Axumin PET is its low specificity for prostate cancer, with studies showing MRI to have a better specificity than Axumin PET (79% vs. 66%) [40]. This is especially true in older patients with benign prostatic hyperplasia (BPH), a commonly observed cause of benign Axumin uptake (Figure 9) [41]. As such, Axumin is not currently FDA approved for prostate cancer staging.

**Figure 10:**
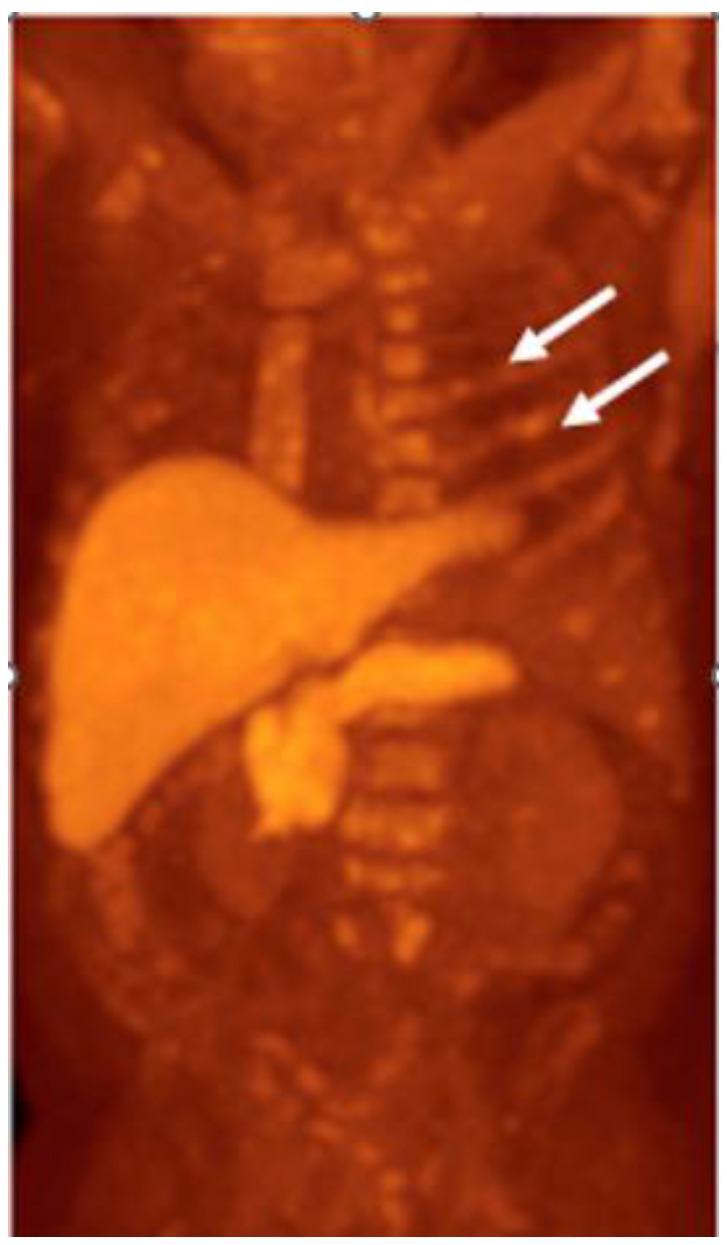
Sixty-seven-year-old male with history of prostate cancer, status post-prostatectomy, now presenting with rising PSA. Axumin MIP image shows multifocal Axumin uptake involving the axial and appendicular skeleton. The heterogeneous and discontinuous pattern of uptake (arrows) favors widespread metastatic disease over a benign diffuse bone marrow process such as treatment-related changes.

**Figure 11:**
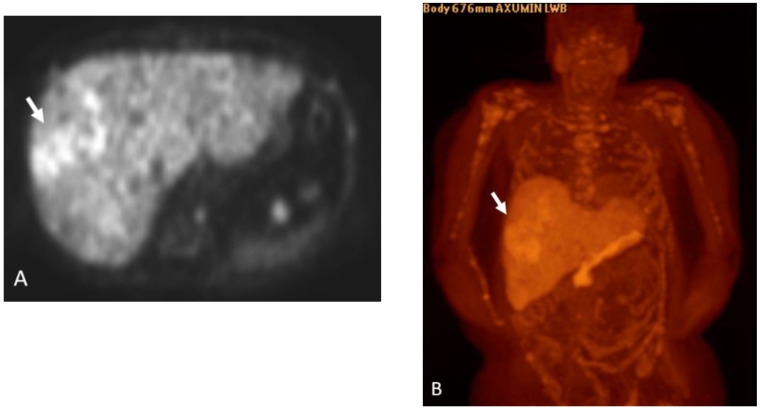
Eighty-year-year-old male with history of prostate cancer, status post-prostatectomy. Axumin PET (**A**) and MIP (**B**) images shows heterogeneous radiotracer uptake in the right hepatic lobe (arrow) concerning for hepatic metastatic disease. Also note the widespread metastatic disease involving the axial and appendicular skeleton.

**Figure 12:**
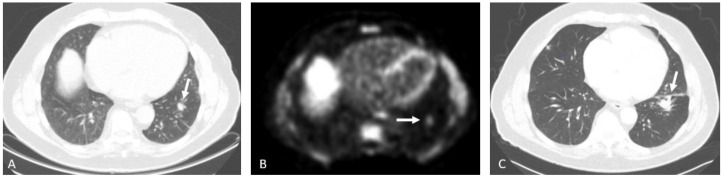
Eighty-four-year-old male with history of prostate cancer, status post-prostatectomy and radiation. NECT (**A**) and corresponding Axumin PET (**B**) images shows a spiculated, solid pulmonary nodule in the left lower lobe (arrows). NECT obtained one year later (**C**) shows the nodule to be increased in size (arrow), further raising suspicion. This was biopsy-proven to be metastatic prostate cancer.

Axumin has high specificity and positive predictive value (96.7% and 95.7%, respectively) when evaluating nodal and osseous metastatic disease in patients with recurrent prostate cancer [42]. Typical hematogenous metastatic spread is usually within the bone, with approximately 90% of patients with metastatic prostate cancer having disease involving and traditionally restricted to osseous metastases (Figure 10) [43]. Other typical sites include the liver, pleura, and lung parenchyma (Figure 11 and Figure 12).

#### 1.9.2. PSMA PET

##### Normal Physiologic Distribution

**Figure 13:**
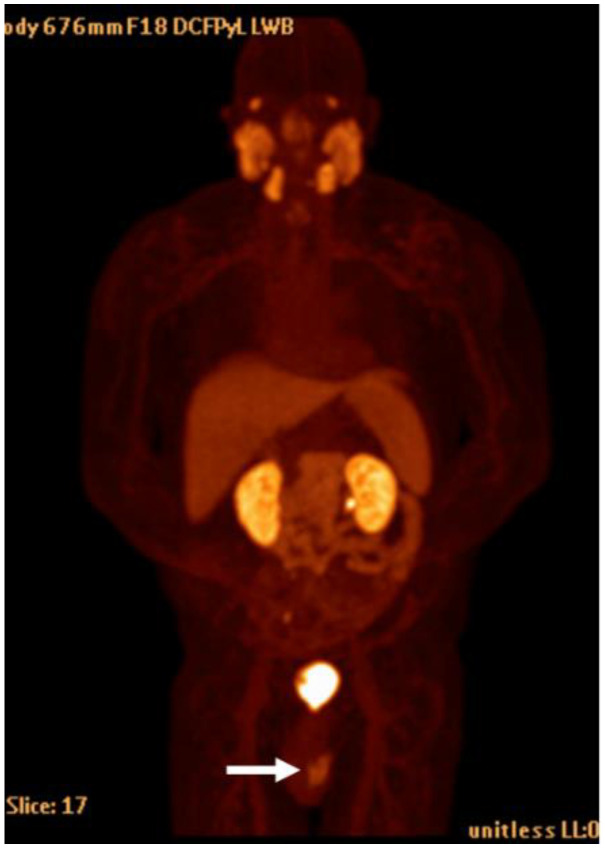
18F DCFPyL MIP image shows normal physiologic uptake within the salivary glands, kidneys and collecting systems, liver, spleen, and bladder. Note the urinary contamination (arrow).

Typical physiologic tracer uptake can be seen within the lacrimal glands, salivary glands, liver, spleen, small intestine, colon, rectum, and kidneys (Figure 13). The normal excretion of radiotracer is through the urinary system, with a small percentage being excreted through the hepatobiliary system. 

For a summary of the physiologic distributions of the PET agents, please see Table 2.

##### Detection of Loco-Regional Disease and Distant Metastases

**Figure 14:**
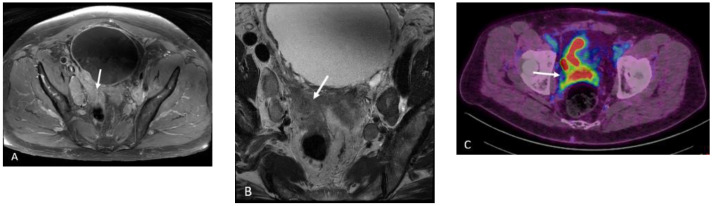
Seventy-nine-year-old male with history of Gleason 9 prostate cancer presenting for initial staging given high risk disease. Axial T1 post-contrast (**A**) and axial T2 (**B**) images demonstrate extra-prostatic extension of tumor into the right seminal vesicle and right pelvic sidewall (white arrows). Axial-fused 18F DCFPyL PET/CT (**C**) demonstrates intermediate- to high-grade activity extending to these regions (white arrows), consistent with miT4 disease.

**Figure 15:**
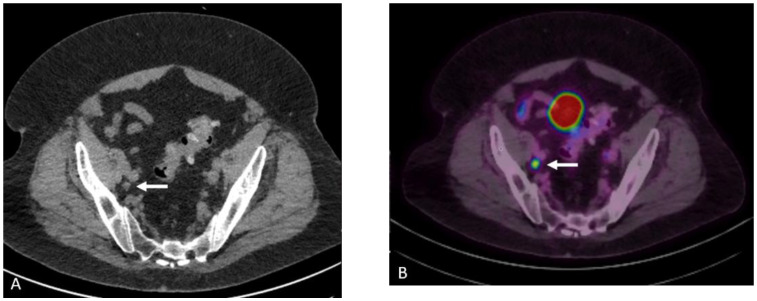
Seventy-seven-year-old male with history of prostate cancer, status post-prostatectomy, now with biochemical recurrence. NECT (**A**) and corresponding fused PET/CT (**B**) images show a sub-centimeter right external iliac lymph node which shows moderate radiotracer uptake concerning for nodal metastatic disease.

**Figure 16:**
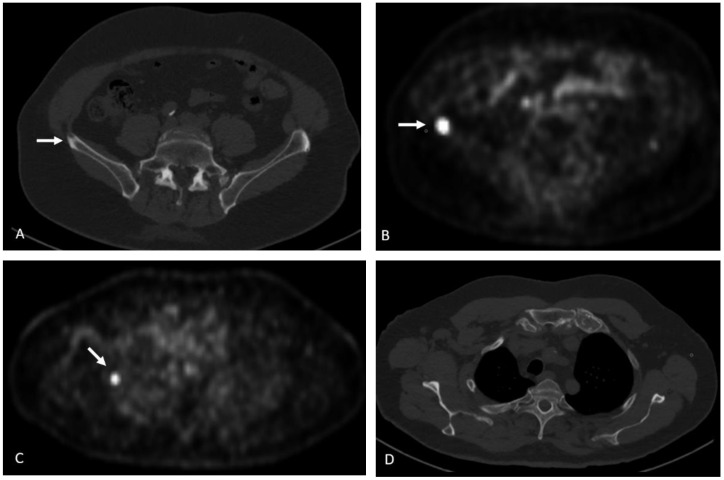
Seventy-four-year-old male with history of prostate cancer, status post-prostatectomy, now with rising PSA levels. NECT at the level of the sacrum (**A**) shows subtle sclerosis of the right iliac crest (arrow). 18F DCFPyL PET image (**B**) shows intense radiotracer uptake in the lesion (arrow). 18F DCFPyL PET image at the level of the scapula (**C**) shows radiotracer uptake within the right lateral 2nd rib (arrow) without a discernable abnormality on CT (**D**).

**Figure 17:**
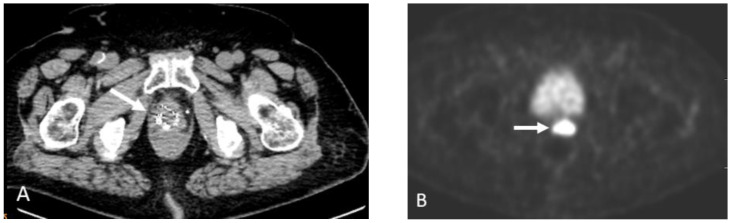
Ninety-two-year-old male with stage IV prostate cancer, status post-brachytherapy, now with biochemical recurrence. NECT image (**A**) shows brachytherapy seeds in the prostate base (arrow) without a discernable abnormality. Corresponding 18F DCFPyL PET image (**B**) shows intense radiotracer uptake in the left prostate base and extending across midline.

While PSMA overexpression in prostate cancer has been known for decades, it has again become a hot topic, with the recent FDA approvals of PSMA-ligand PET agents. A major advantage of PSMA-ligand agents over Axumin is that there is no substantial up-regulation, nor increased radiotracer activity, in the setting of BPH. As such, it has viable uses in localized disease and in guiding biopsy or local treatment (Figure 14) [44]. Beyond localized disease, PSMA has a sensitivity of 81.7%, a specificity of 99.6%, and a positive predictive value of 92.4% when evaluating lymph nodes that are larger than 3 mm (Figure 15) [45]. PSMA was also found to have a superior degree of sensitivity and specificity for osseous metastasis when compared with bone scintigraphy (sensitivity: 100% versus 50%; specificity: 91.7% versus 85.7%) (Figure 16) [46]. This is especially exciting because PSMA PET is multifaceted in its uses, is able to solve multi-organ diagnostic dilemmas with a single scan, and is not limited to evaluating only a small field-of-view (i.e., the pelvis in multiparametric MRI) or bone. PSMA PET is also useful in cases of biochemical recurrence, including assessment for residual or recurrent disease following radiation therapy, and then guiding further therapy (Figure 17) [47]. PSMA PET can also be combined with MRI to improve the sensitivity and specificity relative to either modality individually [48]. 

#### 1.9.3. Pearls and Pitfalls

**Figure 18:**
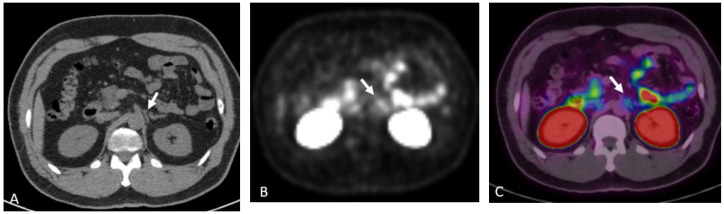
NECT (**A**), 18F DCFPyL PET (**B**), and fused PET/CT (**C**) images show curvilinear soft tissue, which demonstrate low-grade uptake (arrows). The location is characteristic for the celiac ganglion, a normal anatomic structure that can be mistaken for a pathologic lymph node.

**Figure 19:**
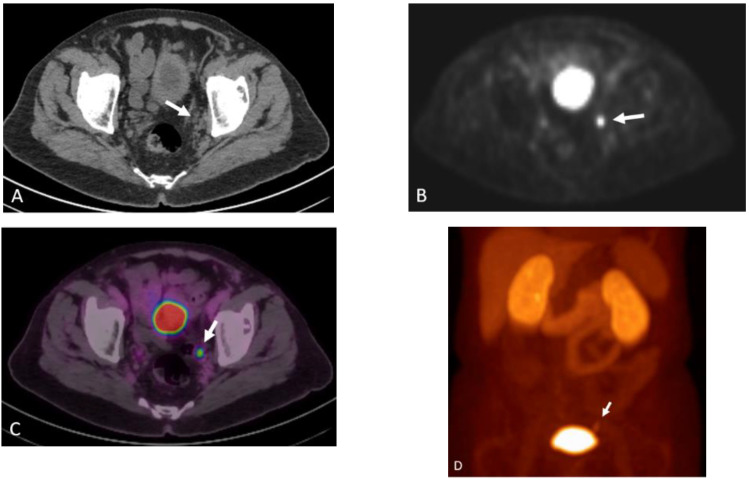
NECT (**A**) shows a round hypoattenuating lesion along the left pelvic sidewall (arrow). Corresponding 18F DCFPyL PET (**B**), fused PET/CT (**C**), and MIP (**D**) images shows that this lesion demonstrates radiotracer uptake. Upon further review, this was found to be excreted radiotracer in the left distal ureter.

**Figure 20:**
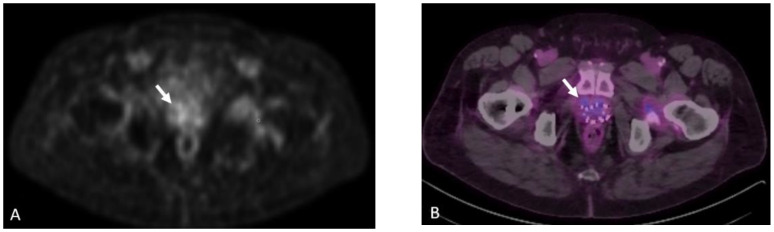
Sixty-four-year-old male with history of prostate cancer, status post-brachytherapy, presenting with rising PSA level. 18F DCFPyL PET (**A**) and fused PET/CT (**B**) images show multifocal radiotracer uptake in the mid-prostate gland (arrows). This uptake subsequently resolved on the follow-up scan (not shown). It was later revealed that the patient had undergone a transrectal prostate biopsy three weeks prior to our study, confirming the uptake to be secondary to inflammation from the recent biopsy.

**Figure 21:**
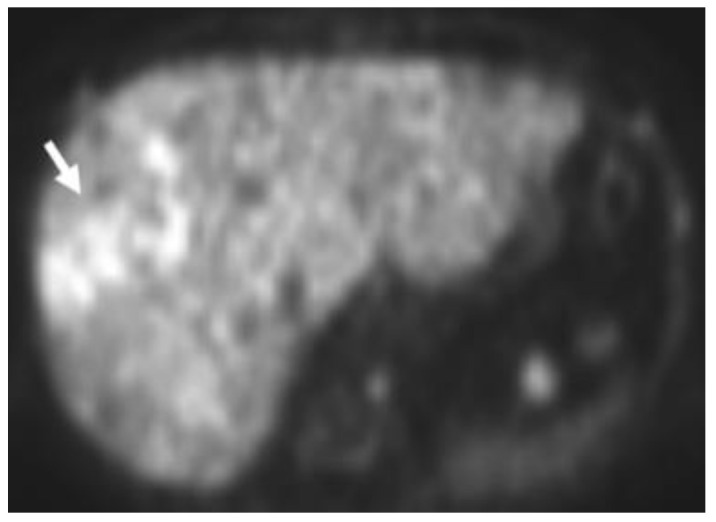
Same patient as Figure 10. Axumin PET image shows physiologic uptake within the liver with more intense uptake in the right hepatic lobe, suggesting metastatic disease. The heterogeneous physiologic uptake pattern in the liver may make identifying small hepatic metastases challenging.

**Figure 22:**
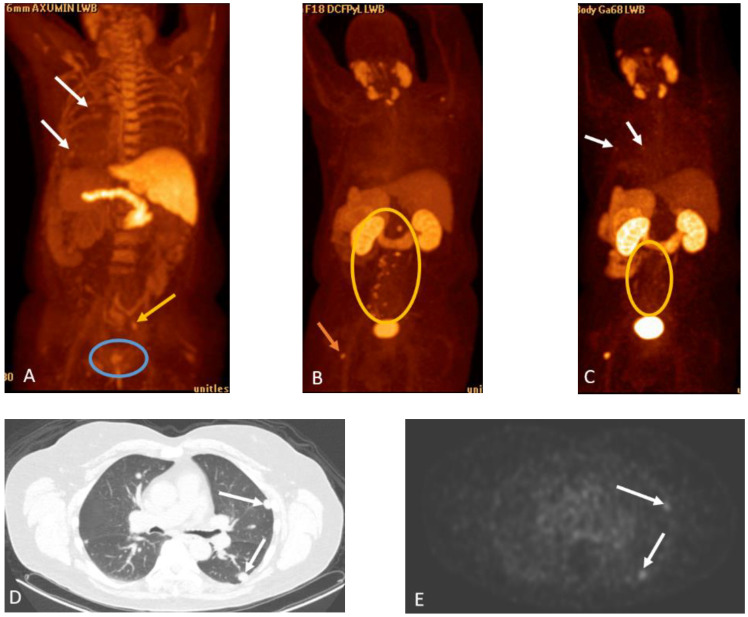
Sixty-eight-year-old man with history of prostate cancer status post-external beam radiotherapy. Initially, he had biochemical recurrence with Axumin PET/CT, (**A**) demonstrating local recurrence in the prostate (blue circle) and a radiotracer avid right pelvic lymph node (yellow arrow). Additionally, mildly radiotracer avid lung nodules were noted (white arrows) with biopsy and subsequent wedge resections, demonstrating multifocal large cell neuroendocrine cancer. Patient was treated with leuprolide acetate therapy and had an initial decrease in serum PSA, followed by an increase, and was imaged with PSMA PET/CT, (**B**) demonstrating resurgent pelvic and retroperitoneal lymphadenopathy and osseous metastases, including the proximal left femur. Following the addition of enzalutamide, he had a decrease in serum PSA, with substantial response on follow-up PSMA PET (**C**, yellow circle). However, there was interval development of multiple low-grade PSMA avid pulmonary nodules (**D**,**E**, white arrows), and biopsy demonstrated recurrent large cell neuroendocrine carcinoma. This case illustrates non-prostate malignancy can display low-grade fluciclovine and PSMA-ligand radiotracer activity.

There are several pitfalls to be aware of when evaluating PET imaging, ranging from normal anatomic variants simulating disease, to benign and malignant non-prostate-related disease uptake simulating metastatic prostate cancer. With imaging technology advances, we have improved our diagnostic ability; however this comes at the cost of increased pitfalls which simulate disease. One such example is the visualization of sympathetic ganglia throughout the body as a result of improved PET image resolution. These ganglia, most commonly the celiac and stellate ganglia, can show low-grade uptake with Axumin and PSMA PET scans in up to 60% of cases [32,49] (Figure 18). Another pitfall with PSMA PET is the urinary excretion of a radiotracer mimicking a lymph node. This is especially true of the distal ureters, as their physiologic location can mimic a pelvic lymph node (Figure 19). This pitfall is much less common with Axumin PET, given its very slow renal excretion and the relatively early timing of the scan. Other confounders of disease include infectious/inflammatory etiologies of radiotracer uptake in the prostate gland, including iatrogenic causes such as biopsies, a fairly common occurrence, given the patient population in question (Figure 20). Due to the relatively high physiologic uptake within the liver, one pitfall of Axumin and PSMA PET scans are that small liver metastases may be missed (Figure 21). Less commonly, radiotracer uptake can be seen in various organs due to a second primary malignancy, and as such, metastatic disease should not be assumed particularly in the setting of a low PSA level (Figure 22).

#### 1.9.4. Future PET Radiotracers

##### 16β-18F—5α-dihydrotestosterone (18-F-FDHT)

**Figure 23:**
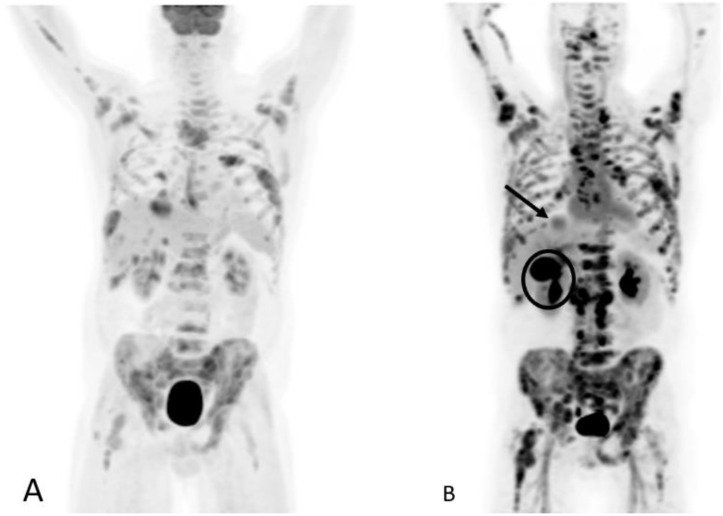
Sixty-one-year-old male with metastatic castration-resistant prostate cancer. FDG PET (**A**) shows widespread metastatic disease primarily involving the osseous structures and lymph nodes, the degree of FDG avidity suggesting high-grade malignancy. F18 DHT PET MIP (**B**) shows greater extent of metastatic disease, including improved visualization of lymph node metastases and a metastatic lesion at the liver dome (arrow). Note the hepatobiliary excretion of DHT with significant gallbladder activity (circle).

16β-18F—5α-dihydrotestosterone (18F-FDHT) is a structural analog of a 5a-dihydrotestosterone (DHT) analog that binds the androgen receptor (AR). Androgen receptors are a transcription factor that are important in castrate-resistant prostate cancer, as these cancers are known to overexpress AR [50,51]. Unlike previously discussed radiotracers, FDHT is less sensitive than 18F-Fluorodeoxyglucose (FDG) PET at detecting prostate metastasis [52]. However, it plays a unique role in assessing treatment with AR receptor antagonists such as Enzalutamide [53]. Another unique use of 18F-FDHT is its role in helping to determine patient prognosis and survival in patients with metastatic castrate-resistant prostate cancer. By comparing 18F-FDG uptake with 18F-FDHT, it was determined that patients who had concordant uptake on both scans had the best prognosis while those who showed significantly greater uptake on 18F-FDG than 18F-FDHT had the worst prognosis (Figure 23) [54].

#### 1.9.5. Miscellaneous Radiotracers

There are several additional PET tracers in the pipeline that show promise for their use in prostate cancer. Gastrin-releasing peptide receptors (GRPR), G protein-coupled receptors, are another useful target for prostate cancer imaging. Bombesin, a 14-amino acid peptide, once labeled to Gallium-68, targets the GRPR receptor as an agonist, and was the first of the Bombesin radiotracers developed for PET imaging [55]. Bombesin receptor antagonists have also been developed and were subsequently found to have better sensitivity than GRPR receptor agonists [56]. One study which looked at the antagonist Gallium-68-RM2 had a sensitivity of 89% when evaluating primary prostate cancer, and a slightly worse sensitivity of 70% when evaluating for nodal metastasis [57]. Another target that has been studied is the serine–protease urokinase-type plasminogen activator (uPA) and its receptor (uPAR). The overexpression of uPAR in tumors has been shown to be associated with aggressiveness and poor prognosis [58,59]. Cell surface protein 6 transmembrane epithelial antigen of prostate 1 (STEAP1) is an androgen-regulated gene that has been the target of numerous antibodies and antibody-drug conjugates. Antibody imaging with Zr 89-2109A PET can be used to measure the effects of antiandrogen therapy on STEAP1 expression, and ultimately treatment efficacy in preclinical studies [60]. 

For a summary of the advances and disadvantages of PET agents, please see Table 3.

## 2. Discussion

PSMA-ligand PET radiotracers have had a massive impact on the clinical management of prostate cancer patients presenting for the initial staging or possible recurrence of known cancer. Despite this, the American Urologic Association (AUA) guidelines still favor bone scintigraphy and either contrast-enhanced pelvic CT or multiparametric MRI (mpMRI) of the pelvis for initial staging in intermediate-to-high risk patients, and PSMA PET “may be obtained”, despite the documented superior performance of PSMA PET [61,62]. We would advocate for the guidelines to better reflect the newest literature and favor PSMA PET for initial staging in intermediate-to-high risk patients. 

Currently, there is active research into further expanding the role of PSMA PET into the screening role prior to diagnosis, either as a primary modality or following a negative/equivocal prostate MRI [63]. The PRIMARY score has been recently described for reporting in the initial diagnosis of prostate cancer via PSMA PET, which showed robust performance [64]. The high sensitivity and specificity of mpMRI and PSMA PET may obviate the need for non-targeted biopsies and reduce the over-diagnosis of prostate cancer that is not likely to be clinically significant (a Gleason score 6 or less) [65].

The wide repertoire and robust diagnostic capability of PSMA PET has forced choline and Axumin imaging into limited supplementary niches. As previously mentioned, an advantage of Axumin over PSMA PET may be its better sensitivity in the detection of local recurrence due to a lack of confounding excreted activity in the bladder, which was demonstrated in a prior study but not in a recent meta-analysis [15,66]. Thus, Axumin may be a second-line option in patients with the biochemical recurrence of previously treated prostate cancer after a negative PSMA PET. Choline has little clinical role currently, with only C-11 being approved for imaging by the FDA, as it is inherently limited by its short half-life, requiring an on-site cyclotron, and it also has a poorer detection rate than PSMA PET [27]. Fluorodeoxyglucose or Dotatate PET can also be considered after a negative PSMA PET to assess for the loss of PSMA expression or neuroendocrine de-differentiation often present in metastatic castration-resistant prostate cancer [67]. 

Despite the promise of PSMA PET, exposure to ionization radiation is a consideration that should be weighed against its benefit. As previously mentioned, the current AUA guidelines still favor bone scintigraphy and contrast-enhanced pelvic CT or mpMRI for the initial staging of intermediate-to-high risk patients. Of the two approaches, combining bone scintigraphy with mpMRI has the lowest effective dose of approximately 4 mSv [68]. While PSMA PET/CT would exceed this dose to approximately 9–10 mSv, PSMA PET/MRI would match the approximately 4 mSv effective dose (varies slightly according to tracer, see Table 1), with the added benefit of superior tissue contrast resolution [69]. As combined PET/MRI machines become more widely available, PSMA PET/MRI is poised to become the gold standard for the assessment of prostate cancer in providing the best equity of ionizing radiation dose and diagnostic capability. 

For the advantages and disadvantages of initial staging modalities, please see Table 4. 

Another caution of PSMA PET is that there is currently limited literature on its assessment of treatment response. Androgen deprivation therapy, commonly used in prostate cancer, is known to upregulate the expression of PSMA [30]. This can result in a pattern of pseudo-progression on imaging, with an increasing radiotracer activity of treated metastases. Indeed, a small series reported this phenomenon to occur in approximately 30% of osseous metastases and 10% of soft tissue metastases [70]. Future clinical trials may need to set rules for the identification of this form of pseudo-progression, to ensure that therapy is not halted prematurely, potentially similar to the “2+2” rule criteria used in the Prostate Cancer Working Group criteria [71].

An assessment of response to target radiotherapy with Lu-177-PSMA-617 is another area that is currently lacking in the literature. As PSMA is a transmembrane protein, it may present an opportunity for the resurgence of agents that target the cytosolic domain, such as In-111 capromab pendetide. These agents would bind preferentially to cells with damaged membranes, and may better depict therapeutic impact, as opposed to agents that bind to viable or damaged cells. The alternate PET agents, including those targeting AR, GRPR, and STEAP1 may also be of some assistance in this era of ever-personalized medicine; however, PSMA seems poised to remain the workhorse agent.

## 3. Conclusions

Prostate cancer is one of the most common causes of mortality in men. Even after definitive treatment with radiotherapy and/or radical prostatectomy, cancer recurrence rates remain high. With traditional CT and MR imaging, it is difficult to accurately localize and to evaluate the extent of disease recurrence. F18 Fluciclovine and PSMA-ligand PET offer a robust solution for the evaluation of recurrent prostate cancer, and in the case of PSMA, they localize disease and guide treatment in intermediate- to high-risk patients. Research involving AR-, GRPR-, and STEAP1-based PET tracers offer the opportunity for imaging-based prognostication, and are an exciting development to look forward to in the future.

## Figures and Tables

**Table 1:** PET agent properties.

PET Agents	Fluorine-18 Fluciclovine	Gallium-68 PSMA-11	18F DCFPyL
**FDA Approval Date**	27 May 2016	1 December 2020	27 May 2021
**Physical Half-Life**	110 min	68 min	110 min
**Mechanism of Action**	Amino acid transport	PSMA binding	PSMA binding
**Patient Preparation**	Avoid exercise for one day prior to the study. NPO for at least 4 h prior to the study.	Gentle hydration and void prior to imaging.	Gentle hydration and void prior to imaging.
**Administered Activity MBq (mSv)**	370 (10)	111–259 (3–7)	333 (9)
**Effective dose (mSv)**	8	1.9–4.4	4.3
**Uptake period**	3–5 min	50–100 min	60–120 min (*package insert suggests >90 min although literature has demonstrated increased lesion detection at delayed time point).
**Acquisition**	-Thighs to vertex. -Preferred injection is the right upper extremity to avoid a false positive Virchow’s node on the left. -Start PET scan 3–5 min after the injection.	-Thighs to vertex. -Preferred injection is the right upper extremity to avoid a false positive Virchow’s node on the left. -Start PET scan 50–100 min after the injection.	-Thighs to vertex. -Preferred injection is the right upper extremity to avoid a false positive Virchow’s node on the left. -Start PET scan 50–100 min after the injection.

**Table 2:** Physiologic distribution of PET agents.

PET Agents	Fluorine-18 Fluciclovine	PSMA-Ligand Agents (18F DCFPyL, Ga-68 PSMA-11)
**Physiologic Distribution**	Pancreas, liver, salivary glands, pituitary glands, small bowel, red marrow, and muscles	Lacrimal glands, salivary glands, liver, spleen, small intestine, colon, rectum, and kidneys

**Table 3:** Reprinted/adapted with permission from (Peter Choyke), (Novel PET imaging methods for prostate cancer; published by World Journal of Urology, 2021). Copyright 2021, Wolters Kluwer.

PET Agents	Advantages	Disadvantages	Comments
11C or 18F Choline	Min urinary excretion availability	Lower sensitivity Non-specific uptake	11C Choline approved by the US FDA
18F FACBC	Improved sensitivity local recurrence	Lower sensitivity Non-specific uptake	18FACBC is approved by US FDA (Axumin)
68Ga or 18F PSMA	High sensitivity High specificity	False positive uptake	Available in the US, Current gold standard
18F DHT	Reports AR activity	Difficult synthesis Noisy scans	Not commercially available
68Ga or 18F Bombesin	High sensitivity	False positive uptake	Not commercially available
18F FDG	Prognostic indicator Widely available	Insensitive in early disease	18F FDG is approved by US FDA

**Table 4:** Advantages and disadvantages of initial staging modalities.

Modality	CT Pelvis and Bone Scintigraphy	Multiparametric Pelvic MRI	PSMA PET/CT	PSMA PET/MRI
Advantages	-Availability and cost	-T staging -No ionizing radiation	-Extrapelvic metastases -N staging	-Superior TNM staging and tissue characterization
Disadvantages	-Extrapelvic non-osseous metastases -Limited specificity of bone scintigraphy -Iodinated contrast exposure	-N staging reliant on morphology -Extrapelvic metastases -Gadolinium contrast exposure	-Less optimal T staging -Ionizing radiation	-Limited availability -Ionizing radiation
Estimated Dose (mSv)	~8–10	0	~12	~4
